# iTRAQ-based high-throughput proteomics analysis reveals alterations of plasma proteins in patients infected with human bocavirus

**DOI:** 10.1371/journal.pone.0225261

**Published:** 2019-11-21

**Authors:** Junmei Bian, Min Liang, Shuxian Ding, Liyan Wang, Wenchang Ni, Shisi Xiong, Wan Li, Xingxing Bao, Xue Gao, Rong Wang

**Affiliations:** Tongren Hospital of Wuhan University (Wuhan Third Hospital), Wuhan, PR China; University of Hong Kong, HONG KONG

## Abstract

Human bocavirus (HBoV) is a member of the genus *Bocavirus*, family *Parvoviridae*, and subfamily *Parvovirus* and was first identified in nasopharyngeal aspirates of Swedish children with acute respiratory tract infection (ARTI) in 2005. It is the causative agent of nasopharyngeal aspirate disease and death in children. The HboV genomic structure is a linear single-stranded DNA (ssDNA). Its clinical pathogenic characteristics have been extensively studied, however, at present the molecular mechanism underlying the pathogenesis of HBoV infection is not completely clear. In this study, a total of 293 differentially expressed proteins (DEPs) between ARTI cases and healthy plasma samples were characterized using isobaric tags for relative and absolute quantitation (iTRAQ)-coupled bioinformatics analysis, among which 148 were up-regulated and 135 were down-regulated. Gene Ontology (GO) and Cluster of Orthologous Groups of proteins (COG) annotated an enrichment of DEPs in complement activation and biological processes like immunity, inflammation, signal transduction, substance synthesis, and metabolism. Kyoto Encyclopedia of Genes and Genomes (KEGG) analysis enriched DEPs mainly in the Wnt signaling pathway (ko04310), PPAR signaling pathway (ko03320), intestinal immune network for IgA production (ko04672), complement and coagulation cascades (ko04610), Toll-like receptor signaling pathway (ko04620) and B cell receptor signaling pathway (ko04662). Further, expression levels of three candidate proteins (upregulated PPP2R1A and CUL1, and downregulated CETP) were validated using western blotting. Our investigation is the first analysis of the proteomic profile of HBoV-infected ARTI cases using the iTRAQ approach, providing a foundation for a better molecular understanding of the pathogenesis of ARTI in children.

## Introduction

Human bocavirus (HBoV) is the causative parvovirus with a linear single-stranded DNA (ssDNA) genome [1, 2] belonging to the family *Parvoviridae* and the subfamily *Parvovirinae* of the genus *Bocavirus*. HBoV can cause respiratory tract diseases and acute gastroenteritis [3], which together are a significant worldwide contributor to morbidity and mortality in children up to age 5 [4, 5]. Since HBoV was first detected in 2005 in children with respiratory infections [1], four different subtypes have been identified (HBoV 1, 2, 3, and 4) [6–8]. HBoV-1 was predominantly detected in the respiratory tract, whereas the three other types (HBoV-2, HBoV-3, and HBoV-4) were mainly identified in stool and associated with gastroenteritis [8]. However, HBoV can also found in asymptomatic people [9]. A genomic study of HBoV suggested a rearrangement of the HBoV group into two classes: human bocaparvovirus 1 (including the previous HBoV-1 and HBoV-3) and human bocaparvovirus 2 (including the previous HBoV-2 and HBoV-4) [10].

HBoV infects individuals worldwide with the average prevalence in respiratory tract samples ranged from 1.0% to 56.8% and in stool specimens from 1.3% to 63% [11]. HBoV is transmitted around the year but infections are enriched during winter and spring [12]. The prevalence of HBoV-1 ranged from 1.5 to 19% in children diagnosed with acute respiratory diseases [13], whereas the prevalence of the three other types ranged from 1% to 26%, 0.4% to 5%, and 0% to 2% in fecal samples from subjects with gastrointestinal illnesses, respectively [14,15]. Clinical symptoms in HBoV-infected patients include rhinitis, pharyngitis, cough, dyspnea, wheezing, pneumonia, acute otitis media, fever, nausea, vomiting, and diarrhea [9]. The coinfection rates of cases with respiratory infections and HBoV-positivity ranges from 8.3% to 100%, and the coinfection ratio is 46.7% relative to gastrointestinal manifestations [16]. Lekana-Douki *et al*. (2018) reported that in 810 nasal swabs and 317 fecal specimens from up to 5-year old children with influenza-like illness or diarrhea in Gabon, 32 (4.4%) and 7 (2.2%) were positive for HBoV [17]. Further, coinfections ratio with at least one other respiratory virus, or intestinal viruses were 84.4% and 42.8%, respectively.

Delays in the diagnosis of infectious disease and the inappropriate use of antibiotics are generally caused by diagnostic uncertainty. Therefore, the development of diagnostic markers for infectious diseases will facilitate more rapid diagnosis and the distinction between infection and non-infectious diseases, leading to improved management, better treatment course and outcomes, and a reduction in the inappropriate use of antibiotics. Proteomics provides for a comprehensive global analysis and is widely used to investigate complex biological functions [18] for identification and characterization of proteins related to pathogenesis and virus-host interactions, contributing to new knowledge of the pathogenesis of various diseases. Two-dimensional gel electrophoresis (2DE) and matrix-assisted laser desorption ionization-time-of-flight mass spectrometry (MALDI-TOF/MS) are widely applied to study the proteomic expression profiles of host cells responding to viral infections, such as severe acute respiratory syndrome [19], H1N1 influenza virus [20], and spleen and kidney necrosis virus [21]. However, 2DE has certain distinct disadvantages, including the ability to identify only a limited number of proteins within a sample and its insensitivity to small and low-abundance proteins. Isobaric tags for the relative and absolute quantitation (iTRAQ) along with nano liquid chromatography mass spectrometry (NanoLC-MS/MS) has emerged as a more powerful quantitative proteomic method because of its high sensitivity and reproducibility, particularly for quantifying low-abundance proteins in the examined samples [21–25].

Knowledge of the pathogenesis of HBoV is very limited, mainly caused by the short of specific cell lines for virus culture or experimental animal models [9]. A deep knowledge of pathogenesis of HBoV is essential for the prevention and treatment of HBoV. Currently, iTRAQ-based proteomics have been widely applied to study differentially expressed proteins (DEPs) of virus-host interactions for understanding the host response to viral infection [22, 24,25]. In the present study, iTRAQ coupled with LC-MS/MS was used to detect and characterize differentially expressed proteins in the plasma of the respiratory tract in patients infected with HBoV and healthy controls. The results will provide novel insight into the regulatory mechanism of HBoV-infected respiratory tract and potential proteins that provide for diagnostic and therapeutic strategies in respiratory tract patients infected with HBoV.

## Materials and methods

### Plasma sample preparation

A total of 10 patients with HBoV-infected acute respiratory tract infection (ARTI) and 10 healthy controls from the 3rd hospital of Wuhan participated in the present study, along with the corresponding patients’ clinical information. Each case group and control group consisted of 6 males and 4 females aged from 9 to 60 months. Each patient provided informed consent, and the study protocol was approved by the Ethic Committee of the 3rd Hospital of Wuhan. Patients with ARTI were diagnosed by two pathologists, who worked independently and were blinded to the present study.

Nasopharyngeal aspirates (NPA) were sampled from 695 children cases that were diagnosed in the Third Hospital of Wuhan from January 2018 to June 2018. The clinical characteristics including disease history, prevalence, symptoms, and signs of nasopharynx inflammation were used for diagnosis, which were confirmed by peripheral blood testing and chest X-ray examinations. Additional protocols including bacterial culture and virus isolation, virus serology, immunofluorescence, an enzyme-linked immunosorbent assay, and hemagglutination inhibition tests were used to further verify diagnostic results when needed.

Viral genomic RNA was extracted from NPA for RT-PCR of nucleocapsid protein gene (VP 1NP 2 gene). Positive specimens were subjected to nucleic acid sequencing that was aligned with known HBoV gene sequence for HBoV-infection confirmation, from which 10 cases were sampled for peripheral blood. Ten patients with bruised knees, elbows, and forehead from January to June 2018 at the 3rd hospital of Wuhan were recruited as controls for sampling of peripheral blood. Medical records for each cases and controls are accessible to identify individual participants during or after data collection.

Peripheral blood from cases and healthy controls was collected and mixed well in EDTA-containing Vacutainers (Becton Dickinson, Franklin Lakes, USA) to prevent clotting. The blood specimens were placed at 4°C for 3–4 h, and centrifuged at 5000 rpm for 5 min. The supernatant was carefully isolated and stored at -80°C. The plasma from the case 1–3, 4–6, and 7–10 were respectively pooled to form three biological replicates BR1, BR2, and BR4 for protein isolation. Similarly, the plasma from the controls 1–3, 4–6, and 7–10 were respectively pooled to form three biological replicates ZC1, ZC4, and ZC7.

### Protein isolation, digestion, and iTRAQ labeling

Protein lysate (7M Urea/2M Thiourea/4% SDS/40 mM Tris-HCl, pH 8.5/1 mM phenylmethylsulfonyl fluoride (PMSF)/2 mM EDTA) was added to an appropriate amount of each sample, mixed well, and incubated for 5 min on ice. We then added dithiothreitol (DTT) at a final concentration of 10 mM, followed by ultrasound in an ice bath for 15 min, and centrifugated for 20 min at 4°C. The supernatant was transferred to a new centrifuge tube, to which we added a 4-fold volume of cold acetone and placed it overnight at -20°C.

The acetone protein precipitant was collected by centrifugation, dried in air, and redissolved in 8 M urea/100 mM triethylamonium bicarbonate (TEAB) (pH 8.0) solution. DTT was added to a final concentration of 10 mM for the reduction reaction in a water bath at 56°C for 30 min, and subsequently added iodoacetamide to a final concentration of 55 mM for an alkylation reaction at room temperature for 30 min in the dark. The Bradford method was used to determine the final protein concentration.

100 μg of protein from each of samples were digested with trypsin. The protein solution was diluted 5 times with 100 mM tetradecyltrimethylammonium bromide (MTEAB), and then we added trypsin at a mass ratio of 1:50 (pancreatin:protein) for enzymatic hydrolysis overnight at 37 °C. The peptide segments were desalted using a C18 column and lyophilized in vacuo.

The peptide was solubilized with 0.5 M TEAB for labelling according to the iTRAQ-8 standard kit (SCIEX) instruction, and mixed for fractionation in Durashell C18 column (5 μm, 100 Å, 4.6 x 250 mm) using an Ultimate 3000 HPLC system (Thermo DINOEX, USA). The peptide segment was separated by increasing the concentration of Acetonitrile (CAN) under alkaline conditions at the flow rate of 1 ml/min. One tube was collected every minute, and a total of 42 secondary fractions were collected and merged into 12 fractions. The merged fractions were desalted on the Strata-X column and dried in a vacuum.

### LC-MS/MS analysis

The mass spectrometry data were captured by TripleTOF 5600 plus liquid chromatography-mass spectrometry (SCIEX) coupled with an Eksigent nanoLC system (SCIEX, USA). The polypeptide sample was dissolved in 2% acetonitrile / 0.1% formic acid, and added to the C18 capture column (5 μm, 100 μm x 20 mm). Gradient elution was performed in a C18 analytical column (3 μm, 75 μm x 150 mm) at a flow rate of 300 nL/min using a 90 min gradient. Two mobile phases were buffer A (2% acetonitrile, 0.1% formic acid and 98% H_2_O) and buffer B (98% acetonitrile, 0.1% formic acid and 2% H2O). For IDA (Information Dependent Acquisition), the 1st-order mass spectra (MS1) were scanned with an ion accumulation time of 250ms, and the 2nd-order mass spectra (MS2) of 30 precursor ions were collected with ion accumulation time of 50ms. MS1 and MS2 spectra were captured in the range 350–1,500 m/z and 50–2,000 m/z, respectively. Precursor ions were excluded from reselection for 15 s.

### Data analysis

ProteinPilot Software (version 4.5, Applied Biosystems, MDS Sciex) was applied to analyze the raw MS/MS file data by searching against UniProt database containing 20,240 sequences (uniprot-swissprot-Homo.fasta) with parameters as followings: the instrument was TripleTOF 5600; cysteine was adjusted with iodoacetamide for iTRAQ quantification; biological qualifications contained ID focus and trypsin digestion; the Quantitate, Bias Correction and Background Correction was examined for protein quantification and normalization. Only proteins with at least one unique peptide and unused value ≥ 1.3 (credibility ≥ 95%) were used for further analysis.

Proteomic data of iTRAQ were quantified using Proteinpilot software (version 4.5, Applied Biosystems, MDS Sciex). The means of pairwise comparisons between biological or technical replicates were first normalized as the ratios, and the minimum ratio was used as p-values to screen the differential expressed proteins (DEPs) by student’s *t* test (two-tailed or unpaired). DEPs were accounted to be significant using *p* < 0.05 and fold changes > 1.5 (upregulated) or < 0.67 (downregulated) as cutoff criteria.

### Gene Ontology (GO), pathway enrichment, and cluster analysis

The DEPs were assigned GO Terms (http://geneontology.org/) to stratify gene products in three groups: molecular function (MF), biological process (BP), and cellular components (CC). The functional annotation of DEGs was performed with Clusters of Orthologous Groups of Proteins System (COG, http://www.ncbi.nlm.nih.gov/COG/) by identifying a cluster of proteins being orthologous across at least three lineages and likely corresponds to an ancient conserved domain. Pathways enrichment analysis was performed by the Kyoto Encyclopedia of Genes and Genomes (KEGG) (https://www.kegg.jp/). Pathway enrichments were statistically examined by Fisher’s exact test, and those with an adjusted *p* < 0.05 were recognized as significant.

### Western blotting analysis

Western blot analysis of the same plasma samples from HBoV-patients and controls was performed to validate proteomic quantitation of the 3 candidate proteins PPP2R1A, CUL1, and CETP. Briefly, plasma samples were diluted 10-fold. Twenty μl of each sample was loaded to membrane, treated with antibodies for PPP2R1A, CUL1, and CETP (1:1000), and exposed for 2 min, 10 min and 10 s, respectively.

## Results

### Protein profile obtained by iTRAQ- coupled LC-MS/MS analysis

**Fig 1:**
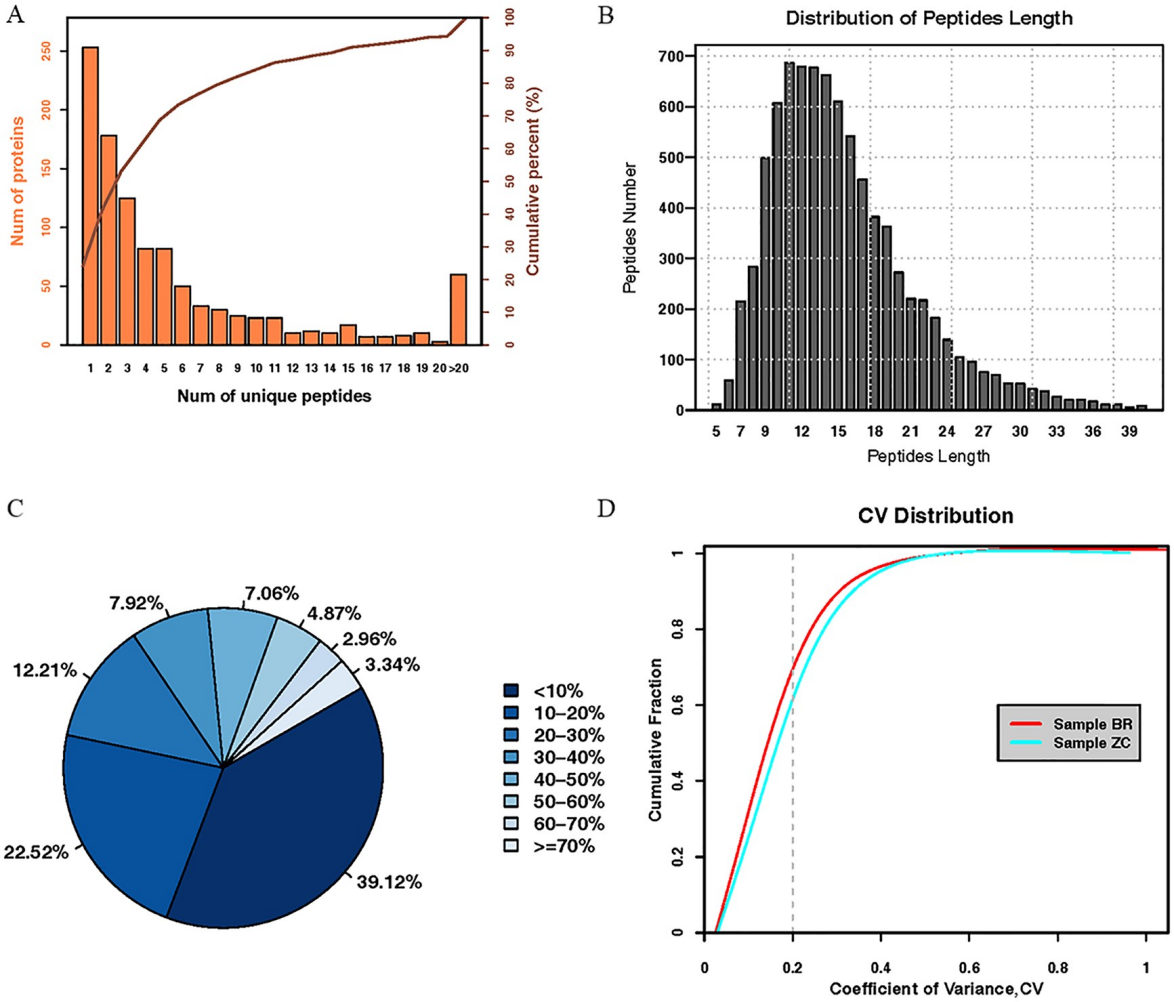
The distributions of unique peptide (A), peptide length and peptide count (B), molecular weight and protein sequence coverage (C), and repeatability analysis (coefficient of variation) (D).

In total, 399625 spectra were identified from the iTRAQ analysis, from which 158700 spectra were matched to known spectra (39.71%), and 8361 were unique spectra, and 1048 were proteins. The distributions of unique peptides showed 795 proteins containing at least 2 unique peptides, accounting for 75.86% of total proteins (Fig 1A). The average length of the peptides was 15.45, and mainly concentrated between 7 and 20 with the 11(Fig 1B). The average coverage of proteins was 21.18%. The percentages of proteins with 0–10% and ≥ 20% of coverage were 39.12% and 38.36%, respectively, indicating a higher credibility of the proteins in the present study (Fig 1C). Repeatability analysis (coefficient of variation) showed 70.12% and 63.80% of the cumulative percentages of CV of the plasma from case and control groups using 20% as the threshold of CV, indicating a higher reproducibility of the plasma samples from the cases (Fig 1D).

**Fig 2:**
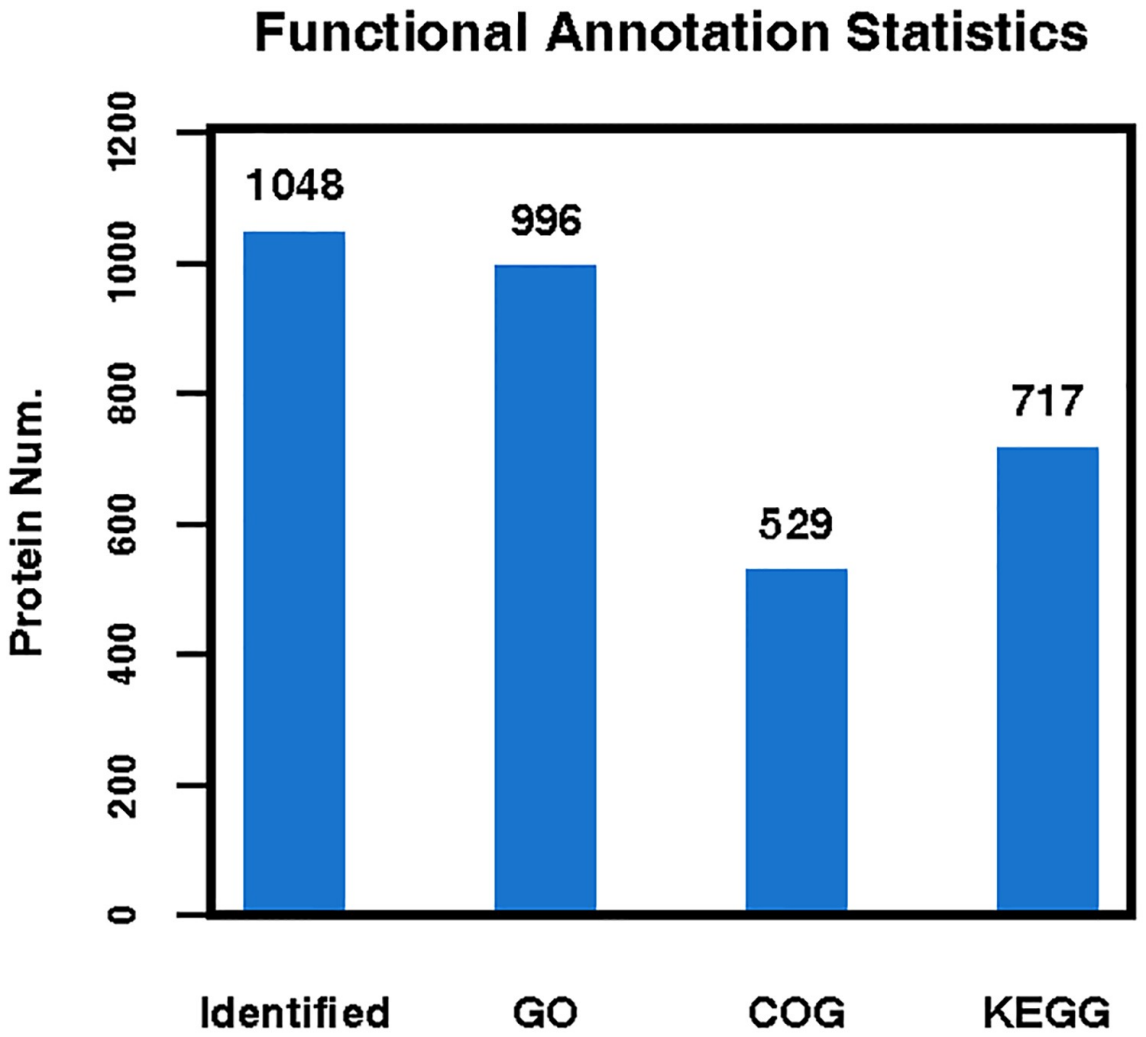
Total proteins identified by iTRAQ and annotated by GO, COG and KEGG analysis.

In total, 1018 DEPs were quantified by iTRAQ coupled with LC-MS/MS analysis, among which 966, 529, and 717 proteins were annotated by GO, COG, and KEGG, respectively (Fig 2). Many proteins were uncharacterized because of limited annotation in the database, and further investigation of their function is needed.

In total, 293 DEPs were characterized, among which 148 DEPs were significantly upregulated, and 135 DEPs were downregulated using a quantitative ratio over 1.5 (fold change > 1.5 or < 0.67) and a *p* < 0.05 as cutoff criteria.

### Functional classification of DEPs

**Fig 3:**
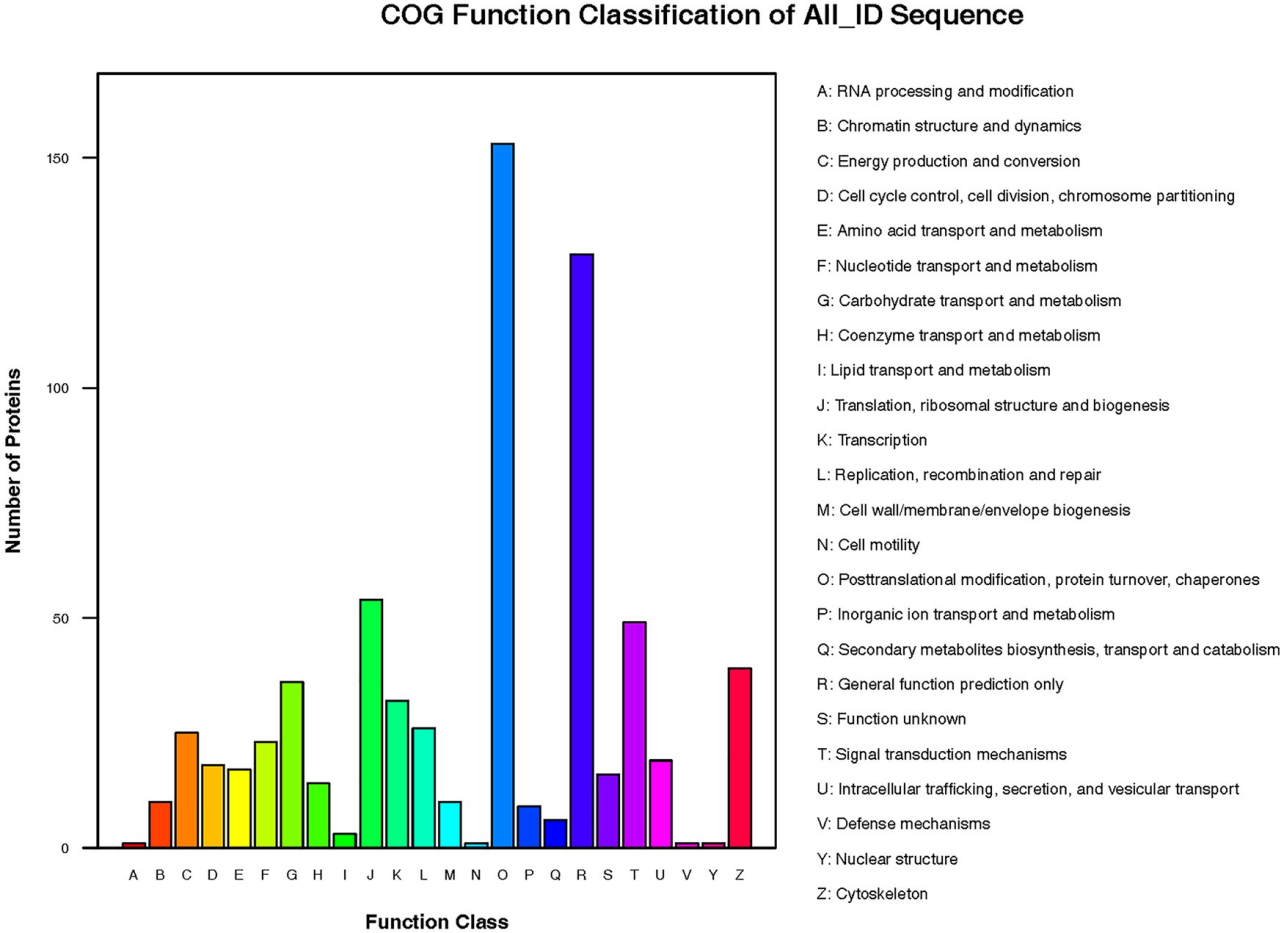
COG function classification of the DEPs.

These 293 DEPs were assigned to 24 groups according to their function as listed in the COG annotation. The top two groups were post-translational modification and general function prediction only, followed by translation, ribosomal structure and biogenesis, and signal transduction mechanism (Fig 3).

**Fig 4:**
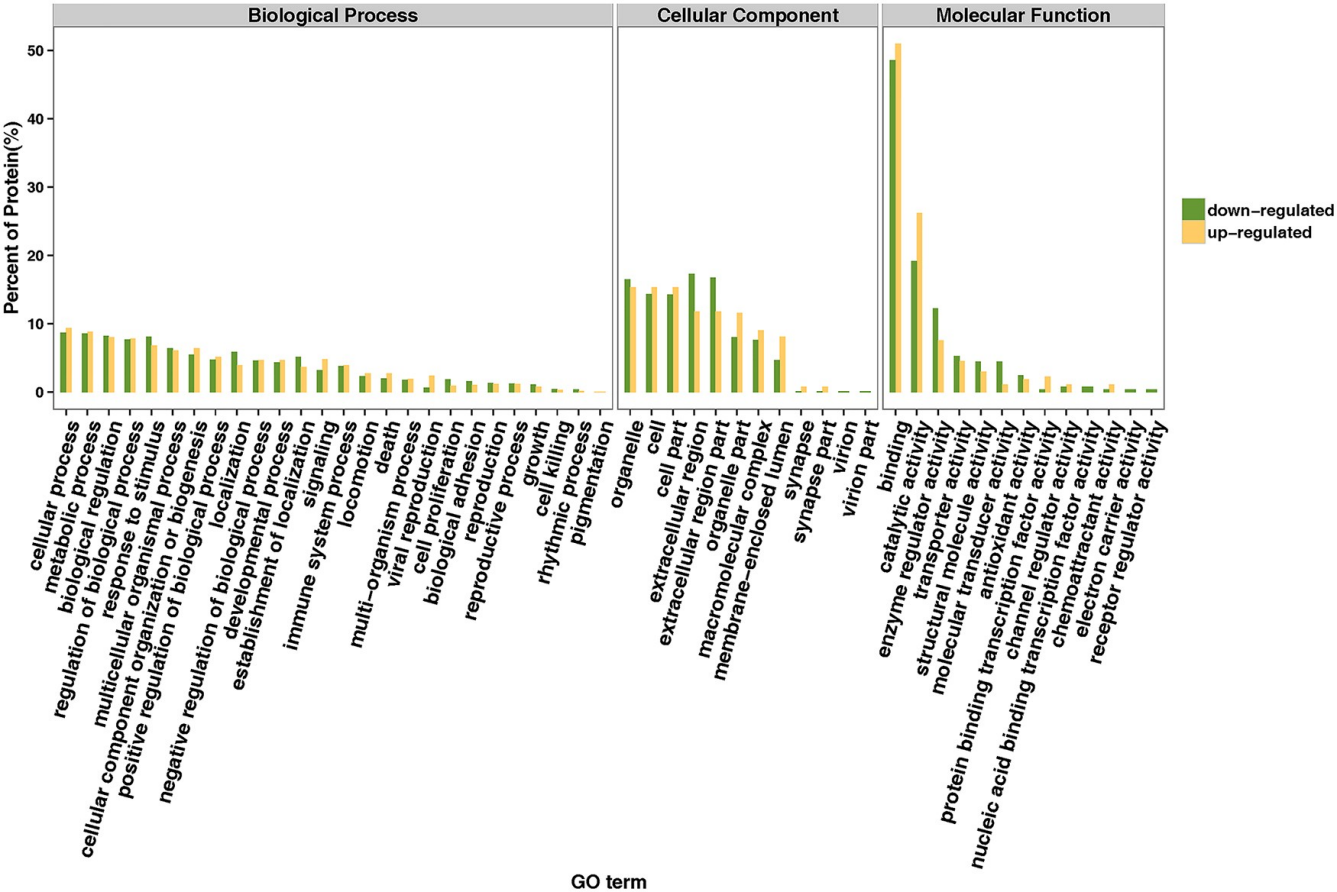
GO function of the DEGs. Proteins were clustered into three main categories: CC, BP, and MF. The y-axis represents the percentage of a specific category of proteins in each main category.

DEPs were assigned to Biological Process(BP), Molecular Function(MF), and Cellular Component(CC) (Fig 4 and S1 File). For the BP category, DEPs were well distributed in different biological processes, and the major functional categories were cellular process (9.94%), metabolic process (8.84%), biological regulation (8.17%), regulation of biological process (7.84%) and response to stimulus (7.25%). In the MF category, DEPs were mainly assigned to binding (51.59%), catalytic activity (23.92%), enzyme regulator activity (7.60%) and structural molecule activity (4.48%). In the CC category, DEPs were mainly enriched in cell part (16.30%), organelle (15.97%), extracellular region (12.90%) and extracellular region part (12.64%). Significant differences in GO functional classifications were observed between upregulated and downregulated DEPs. As shown in Fig 2, downregulated DEPs had two unique GO terms- electronic carrier activity and receptor regulator activity in the CC category compared with upregulated DEPs. There were also significant differences in the percentage of DEPs assigned to the same functional terms between upregulated and downregulated DEPs.

**Fig 5:**
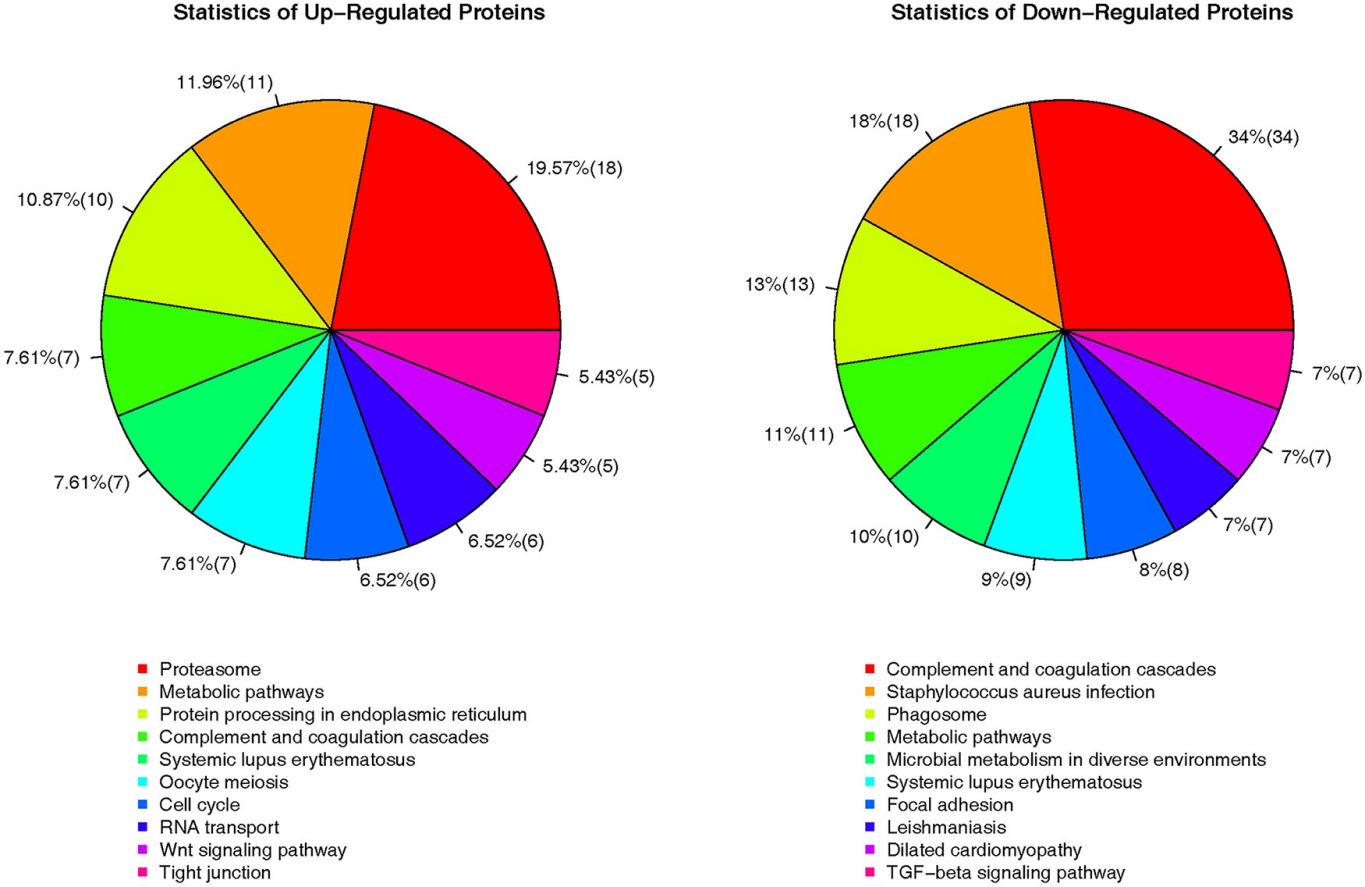
KEGG pathway analysis of the upregulated and downregulated DEGs.

KEGG analysis was also applied using the Database for Annotation, Visualization, and Integrated Discovery (DAVID) (https://david.ncifcrf.gov/) to functionally enrich gene functions and to identify functional and metabolic pathways. The upregulated DEPs were mainly enriched in the proteasome (18) and metabolic pathways (11). The pathways associated with downregulated DEPs were related to complement coagulation cascades (34), staphylococcus aureus infection (18), phagosome (13), and metabolic pathways (11) (Figs 4 and 5, S1 File). Four functions including metabolic pathways, systemic lupus erythematosus, microbial metabolism in diverse environments, and glycolysis/gluconeogenesis were common in the top 10 pathways enriched from both downregulated and upregulated DEPs.

### Validation of protein identification and quantification by western blot

**Fig 6:**
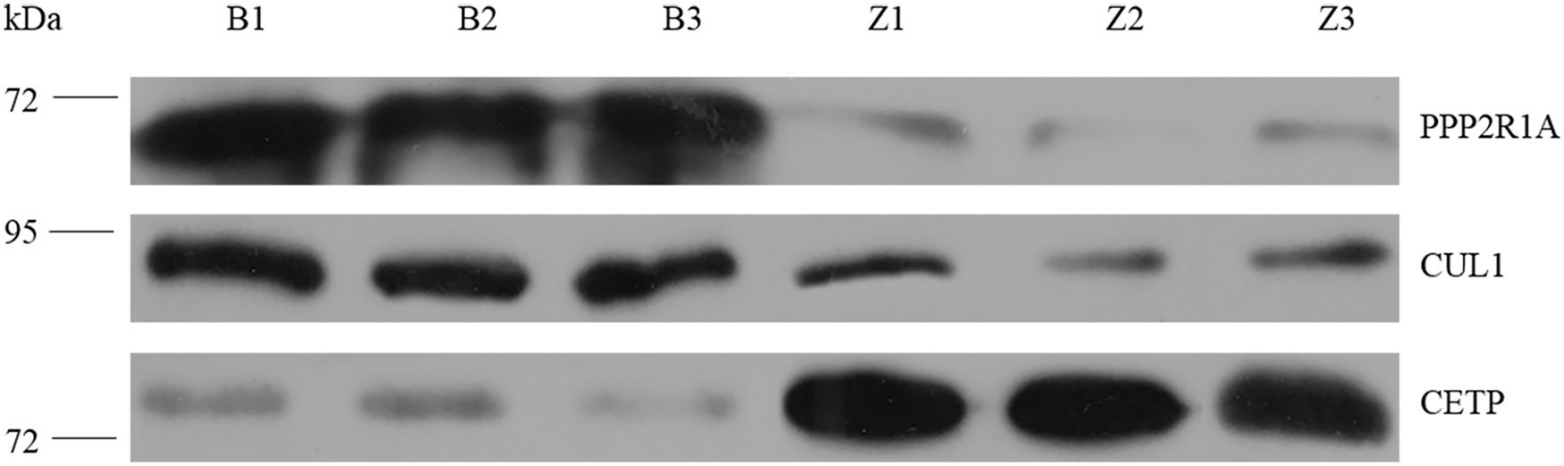
Validation of 3 DEPs by western blot analysis. Top, PPP2R1A antibody test (65kd). Middle, CUL 1 antibody test (90kd). Bottom, CETP antibody test (75kd).

Three candidate proteins (upregulated PPP2R1A and CUL1, and downregulated CETP) were validated by western blotting (Fig 6) to confirm the results of iTRAQ-labeled LC-MS/MS analysis. The results of western blotting are consistent with those determined from iTRAQ.

## Discussion

From the standpoint of host cell proteins, interactions between a virus and its host are highly complicated processes, frequently resulting in abundant alterations in the expression of signaling pathway proteins [26]. A set of proteomic approaches have been widely used to investigate viral-host interactions [22, 24, 25, 27, 28] for a better understanding of pathogenesis. In the present study, we performed iTRAQ combined with LC-MS/MS analysis in the plasma of respiratory tract patients infected with HBoV. In total, 1048 proteins were determined, of which 148 DEPs were significantly upregulated and 135 downregulated. We then validated three candidate proteins by western blotting analysis, which supported our iTRAQ results. These DEPs were associated with a lot of biological processes such as complement activation and biological processes including immunity, inflammation, signal transduction and regulation, substance synthesis, metabolism metabolic pathways, protein processing in endoplasmic reticulum and protein turnover mediated by the proteasome. They were enriched the in Wnt signaling pathway (ko04310), peroxisome proliferator-activated receptor (PPAR) signaling pathway (ko03320), intestinal immune network for IgA production (ko04672), complement and coagulation cascades (ko04610), Toll-like receptor(TLR) signaling pathway (ko04620), and B cell receptor signaling pathway (ko04662) (S1 File). The profiles of the present study provide a foundation for the development of diagnostic biomarkers and therapeutic schemes, and the identified DEPs provide novel insight into the potential pathological mechanism elucidating HBoV infection.

PPP2R1A (Protein Phosphatase 2, Regulatory Subunit A Alpha), belonging to the PPP family of phosphatases, is participated in negative regulation of a variety of vital cellular processes such as cell growth and division. Cullin 1 (CUL1) is a human protein from cullin family, and acts an important role in protein degradation and protein ubiquitination. Both PPP2R1A and CUL1 belong to immune response genes [29], and play a crucial role in various cellular activities during HBoV infection as a host restriction factor to suppress transcription in astrocytes. In the present study, viral-related proteins PPP2R1A and CUL1 were unregulated to modulate the Wnt signaling pathway (ko04310) and transforming growth factor-β (TGF-β) signaling pathway(ko04350) (S1 File), consistent with previous results [29–31]. Wysocki *et al*. (2012) discovered upregulated PPP2R1A during PRRSV infection [29]. CUL1 was upregulated to modulate cell multiplication, migration, invasion and metastasis [30, 31]. Wnt signaling pathway is a critical pathway in cell cycle control, whereas TGF- β signaling pathway plays a critical role in cell signaling regulation of multiple vital processes including multiplication, discrimination, apoptosis, EMT, and migration. TGF- β signaling pathway was demonstrated to being a doubtful advantage in normal and pathogenic development, especially in organ fibrosis, vascular disorders, and carcinoma. Previous studies reported the dysregulation of the Wnt signaling pathway during pseudorabies virus (PRV) infection [24] and the enhanced influenza virus mRNA and virus production by triggering Wnt pathway [32]. The reaction to EBOV infection was regulated by TGF- β signaling pathway[33], and the improved secretion of TGF-β1 and vascular endothelial growth factor (VEGF) in EBOV-infected cells were improved by temporally upregulating TGF-β-modulated signaling responses. TGF-β that is secreted from host cells is a latent and non-acting complex, and triggered by the neuraminidase (NA) of influenza A virus to modulate its signaling function [34]. The quantity of active TGF-β was increased in the culture medium, and partial cells with high quantity of the integrin subunit α5 and fibronectin infected by influenza A [35]. Particularly compared with binding to mannose the adhesion of bacteria to fibronectin was also improved[35]. TGF-β signaling was interfered with viruses in many ways, such as adjustment of TGF-β protein expression and TGF-β receptors or SMADs factors. Further study is needed to elucidate TGF-β-regulated mechanism in interfering with HBoV infection.

Protein turnover is a highly regulated bioprocess managing infinite multitude pathways for maintaining cellular homeostasis and modulating the cellular environment [36]. Cellular growth control was affected by mass protein degradation with proteasome to cause aberrant cell cycle progression, transcription, DNA repair, apoptosis, and angiogenesis [37]. Proteasome is functioned as a crucial element in the reproduction of many viruses, and in antiviral immune defenses [38]. Proteasome-dependent, ubiquitin-independent degradation is highly observed during viral infections. In the present study, 18 DEPs were enriched in proteasome function catergory. Interestingly, all of the 18 proteins were upregulated. Among them, CUL1 was involved in ubiquitin-mediated proteolysis (ko04120) (S1 File). Ubiquitin molecules altered covalently substrates targeted by that proteasome [16]. As a 76 amino acid protein ubiquitin covalently linked to substrates at N-terminal or lysine residues through its C-terminal amino acid and then added ubiquitin molecules to one of seven lysine residues for assembling ubiquitin chains. Polyubiquitin chains were affixed the targeted substrates to the proteasome by direct interplay between Rpn13 and Rpn10 subunits of the 19S RP [39] in leading covalently marked proteins for proteasomal degradation. Further study is needed to elucidate potential functions of these upregulated DEPs during HBoV infection.

Cholesterol ester transfer protein (CETP) is a glycoprotein connecting to high-density lipoproteins (HDL) in blood and engaging in the movement of neutral lipids such as cholesteryl ester and triglyceride amongst lipoprotein particles. Biological function of CETP is not fully understood, however, CETP is involved in regulation of PPARs signaling pathway (ko03320) and TLRs signaling pathway (ko04620), thus playing key anti-inflammatory roles for protein family including bactericidal permeability increasing protein (BPI). Germline-encoded pattern recognition receptors (PRRs) regulates the host intrinsic immune system for intrinsically defensing pathogen attacks PRRs were demonstrated to distinguish host segments from pathogens through identification of conserved regions within a group of pathogens to activate signaling cascades for terminating the expression of antimicrobial products, inflammatory cytokines, and chemokines [40]. Among three families of PPRs (TLRs, RIG-I-like receptors (RLRs), and nucleotide binding-oligomerization domain (NOD)-like receptors (NLRs) [41], TLRs act as essential molecules of the host intrinsic immune system to sense the conserved molecular signatures within a group of pathogens and trigger intrinsic immune responses to pathogen attacks. Numerous TLRs were proven to involve in the early interaction between host cells and attacking pathogens in coordination with viral reproduction and/or host responses to finally affect viral pathogenesis. Pathogens have developed approaches for escaping or defecting signaling through the TLR pathways for survival and replication. PPARs belong to the nuclear receptor superfamily including PPARα, PPARβ/δ and PPARγ, and critically regulate cellular differentiation and development, metabolism, and tumorigenesis [42]. The expression of genes involved in lipid metabolism, adipogenesis, maintenance of metabolic homeostasis, and inflammation was modulated by heterodimers of PPARs—retinoid X receptor (RXR) heterodimers to induce anticancer impacts in various tumors. PPAR activity was transcriptionally regulated by nongenomic cross-talking with phosphatases and kinases such as ERK1/2, p38-MAPK, protein kinase A (PKA), protein kinase C (PKC), 5' AMP-activated protein kinase (AMPK), and glycogen synthase kinase-3 (GSK3). In the present study, CETP was downregulated during HBoV infection, potentially representing a novel pharmacological target for HBoV treatment.

Energy and small molecule metabolites such as adenosine triphosphate (ATP), nicotinamide adenine dinucleotide (NADH), nicotinamide adenine dinucleotide phosphate (NADPH), and carbon are essential for the multiplication of virus in host cells [43], among which pyruvate is a core molecule for various forms of eukaryotic and human metabolism. As the end-product of glycolysis, pyruvate in the cellular cytoplasm is preprogramed being a major fuel input for delivery into mitochondria to support citric acid cycle carbon flux as Several enzymes such as newly reported mitochondria pyruvate carrier, pyruvate dehydrogenase, and pyruvate carboxylase participated in the regulation of mitochondrial pyruvate metabolism for overall pyruvate carbon flux. In the present study, 22 DEPs were classified into metabolic pathways, among which 11 DEPs were upregulated and 11 were downregulated. These proteins mainly participated in glycolysis and pyruvate metabolism, demonstrating that HBoV used energy and small molecule metabolites from the host cell for multiplication by hijacking host cell metabolic processes. The understanding of the regulatory mechanisms and critical proteins in glycolysis and pyruvate metabolic pathways after HBoV infection might provide a potentially novel targets for antiviral drug discovery and therapeutic development through metabolic pathway inhibitors.

The endoplasmic reticulum (ER) is on many molecular chaperones for protein folding and assembly [44] is very important for protein synthesis and maturation. ER was poisoned by a protracted enormous increasing of free calcium levels in the cytoplasm and mitochondria, leading to the severe form of stress on traget cells. Therefore, ER dysfunction was mainly caused by toxic accident that generated in the pathogenic development to block vital ER responses or ER calcium pool has been confirmed being one of the principal targets of toxic metabolites or intermediates such as oxygen free radicals produced during the pathogenesis of acute disorders and degenerative diseases. ER dysfunction was mainly caused by toxic intermediates generated in pathogenic development to block vital ER reactions or fully activation of unfolded protein response (UPR) and ER-associated protein degradation (ERAD). The entire UPR plays an essential role in regulation of x-box-binding protein 1 (XBP1) function as a transcription factor during ER stress. However, the reaction to ER stress is blocked when XBP1 protein synthesis is interrupted to certain level. ER stress is further aggravated by triggering ER- (ERAD) to deteriorate unfolded proteins through the ubiquitin/proteasomal pathway that is defective in acute disorders and degenerative diseases. In the present study, 10 DEPs participated in protein processing in the ER, consistent with the results of previous studies [45, 46], which revealed facilitation of viral replication by ER alternations and activation of the unfolded protein response with viral infections. Interestingly, all of the 10 proteins were upregulated, including the heat shock proteins 71 kDa, 105 kDa (hsph1), and hsp90-alpha and beta-1(S1 File). Heat-shock response activation was thought to play a virus-specific function assuring appropriate protein synthesis by facilitating protein folding. Previous studies confirmed the involvement of Hsp90 in the assembly and nuclear transfer of viral RNA polymerase subunits and facilitation of viral replication in various virus including HIV-1[47], ebola virus [48] and rotavirus [49]. Therefore, Hsp90 might be a potential target inhibitor for preventing viral infections [50]. Other upregulated ER-stress proteins including calreticulin and calnexin important for calcium storage and protein folding [51] were shown to act crucial role in viral infections [52, 53]. Further study is needed to elucidate the functions of these proteins in preventing HBoV infection.

In conclusion, the findings of the present study provide a comprehensive knowledge of the proteomic profiles of HBoV-infected ARTI patients using iTRAQ-based quantitative proteomics. In total, 293 proteins were significantly altered, among which 148 were upregulated and 135 were downregulated. These preliminarily proteomic results are needed for further validation to confirm the functional roles of these proteins in the pathogenesis of HBoV, thereby enabling novel antiviral therapeutic targets of HBoV infection.

## Supporting information

S1 File The molecular characterization of the DEPs by GO and KEGG analysis. (XLSX)

S1 Raw Images The original uncropped and unadjusted images underlying all blot or gel results. (DOCX)
