# Supplementary figures and images for: iTRAQ-based high-throughput proteomics analysis reveals alterations of plasma proteins in patients infected with human bocavirus
Source: PLoS One. 2019 Nov 21;14(11):e0225261. doi: 10.1371/journal.pone.0225261 (PMC6872134; doi:10.1371/journal.pone.0225261)

PPP2R1A


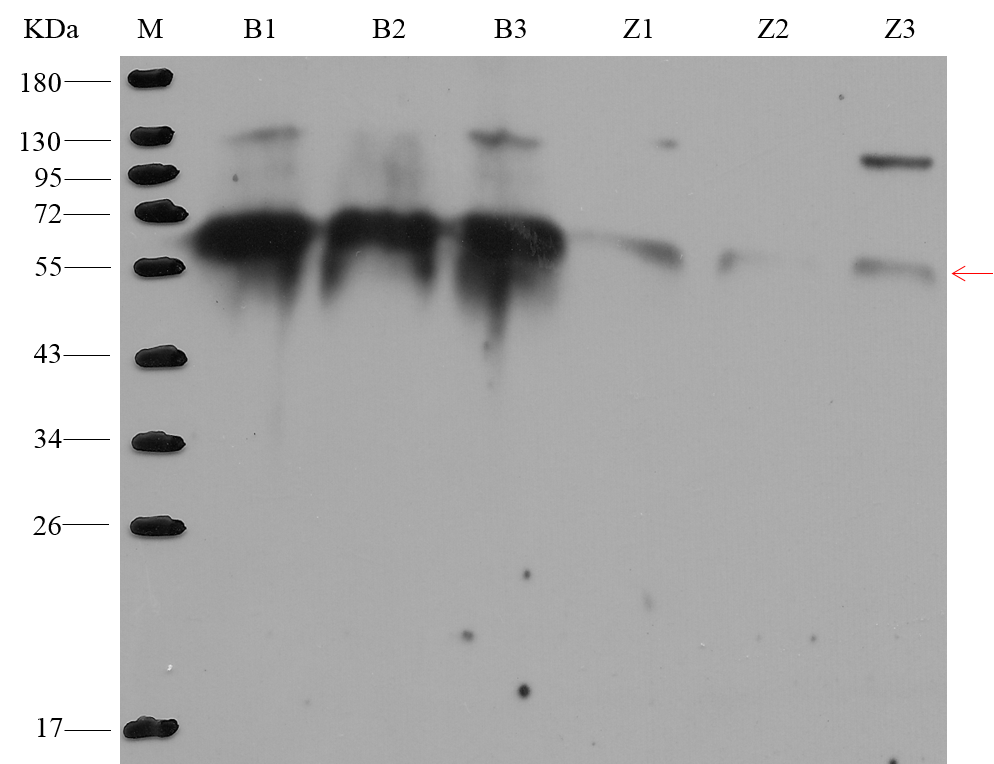


CUL1


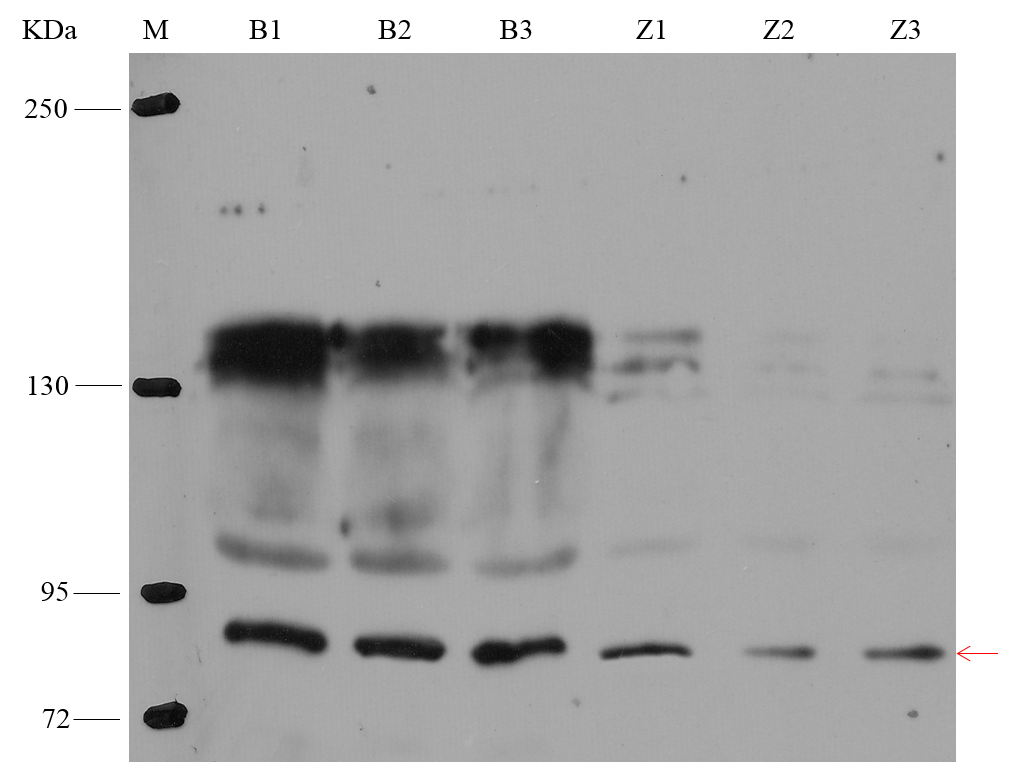


CETP


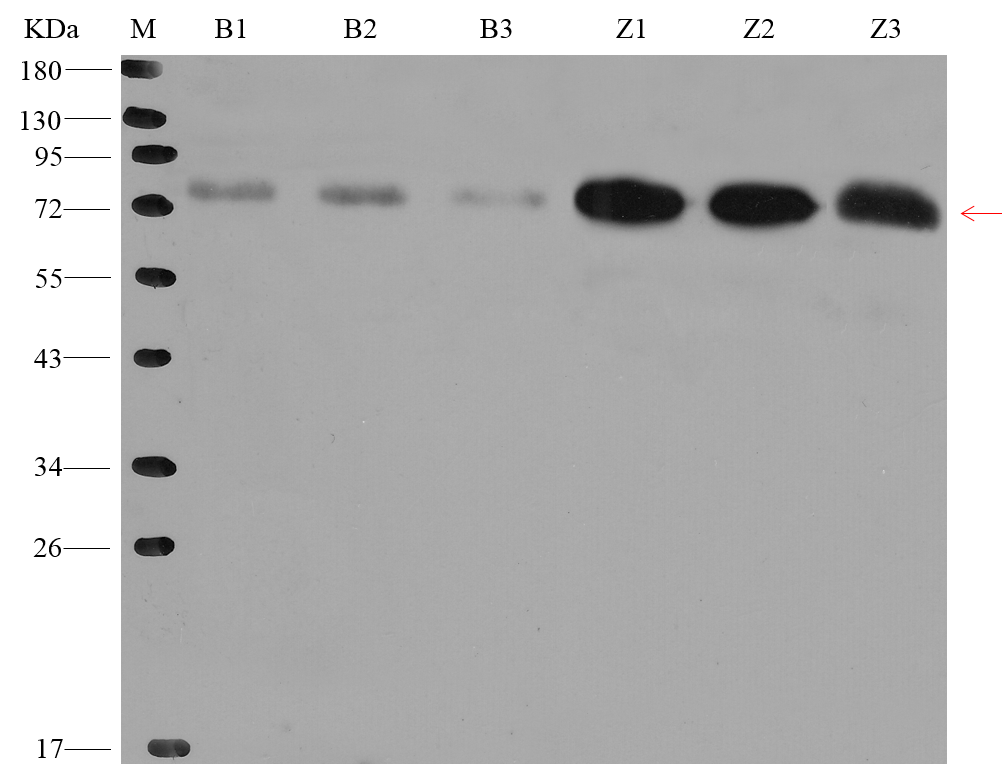

Supplement: S1 Raw Images — (DOCX) [file pone.0225261.s002.docx]
